# Thermally Conductive Molten Salt for Thermal Energy Storage: Synergistic Effect of a Hybrid Graphite‐Graphene Nanoplatelet Filler

**DOI:** 10.1002/gch2.202300053

**Published:** 2023-08-31

**Authors:** Adi Lavi, Avia Ohayon‐Lavi, Yelena Leibovitch, Shmuel Hayun, Efrat Ruse, Oren Regev

**Affiliations:** ^1^ Department of Chemical Engineering Ben‐Gurion University of the Negev Beer‐Sheva 84105 Israel; ^2^ Department of Chemistry Nuclear Research Center‐Negev P.O.B. 9001 Beer‐Sheva 84190 Israel; ^3^ Department of Materials Engineering Ben‐Gurion University of the Negev Beer‐Sheva 84105 Israel

**Keywords:** exfoliation, graphene, molten salt, Raman mapping, thermal conductivity

## Abstract

Renewable energy technologies depend, to a large extent, on the efficiency of thermal energy storage (TES) devices. In such storage applications, molten salts constitute an attractive platform due to their thermal and environmentally friendly properties. However, the low thermal conductivity (TC) of these salts (<1 W m^−1^ K^−1^) downgrades the storage kinetics. A commonly used method to enhance TC is the addition of highly conductive carbon‐based fillers that form a composite material with molten salt. However, even that enhancement is rather limited (<9 W m^−1^ K^−1^). In this study, the partial exfoliation of graphite to graphene nanoplatelets (GnP) in a molten salt matrix is explored as a means to address this problem. A novel approach of hybrid filler formation directly in the molten salt is used to produce graphite–GnP–salt hybrid composite material. The good dispersion quality of the fillers in the salt matrix facilitates bridging between large graphite particles by the smaller GnP particles, resulting in the formation of a thermally conductive network. The thermal conductivity of the hybrid composite (up to 44 W m^−1^ K^−1^) is thus enhanced by two orders of magnitude versus that of the pristine salt (0.64 W m^−1^ K^−1^).

## Graphical abstract

Thermal conductivity (TC) enhancement of a molten salt, used for thermal energy storage (TES), is achieved by partial exfoliation of graphite to graphene nanoplatelets (GnP) during the thermal treatment in molten salt. The well‐dispersed GnP and graphite in the molten salt form a thermally conductive network that enhances the TC of the hybrid filler‐salt composite by two orders of magnitude.

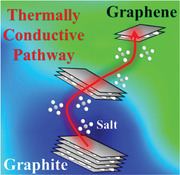

## 1 Introduction

The increasing global demand for energy continues to catalyze immense environmental damage and hence to create an urgent worldwide need for alternative, renewable energy sources, particularly in light of the fact that the Earth's resources of fossil fuels are finite.^[^ ^1^, ^2^ ^]^ In the field of renewable energy technologies, solar energy is perhaps the most promising,^[^ ^1^, ^2^, ^3^, ^4^, ^5^, ^6^, ^7^ ^]^ with the concentrated solar power (CSP) technology being of special interest, since it supplies dispatchable energy by converting heat stored in integrated thermal energy storage (TES) devices.^[^ ^4^, ^8^, ^9^, ^10^, ^11^ ^]^ The different types of TES systems include those based on sensible heat, latent heat, or thermochemical reactions, where sensible heat storage (SHS) exploits heat capacity to change the temperature of the storage medium, latent heat storage (LHS) exploits the heat generated during a phase change, and thermochemical systems employ energy generated during the breaking and re‐forming of molecular bonds.^[^ ^12^ ^]^

Despite progress in the past decade, the efficient use of solar energy still requires the development of advanced technologies and materials for the storage (daytime) and release (nighttime) of thermal energy.^[^ ^5^, ^6^ ^]^ Among the technologies applied commercially in CSP plants are those using molten salts.^[^ ^3^, ^7^, ^8^, ^9^, ^10^, ^13^, ^14^, ^15^, ^16^ ^]^ Molten salts have the potential to provide a versatile platform for TES applications in SHS and LHS systems due to their extensive liquidus temperature (250–1680 °C) and heat of fusion (68–1041 J g^−1^)^[^ ^17^ ^]^ ranges, and they have indeed been used industrially as heat transfer fluids (HTF) in thermo‐solar applications.^[^ ^18^, ^19^ ^]^ In contrast to other HTFs, such as thermal oils, molten salts exhibit the advantages of high density, high specific heat capacity, good chemical and thermal stability, low viscosity, and low vapor pressure.^[^ ^19^ ^]^ In addition, they are cheaper, environmentally friendly, and non‐flammable.^[^ ^18^ ^]^ The above notwithstanding, molten salts have the inherent disadvantage of low thermal conductivity (TC) (<1 W m^−1^ K^−1^),^[^ ^20^ ^]^ which impairs their heat transfer performance and hence limits their efficient use in TES applications.

Efforts to improve the TC of molten salt matrices have included the addition of high TC materials, termed fillers, such as graphene or graphite (≈4000 and ≈1000 W m^−1^ K^−1^, respectively),^[^ ^17^, ^21^, ^22^ ^]^ to form a composite material.^[^ ^23^, ^24^, ^25^, ^26^ ^]^ Graphene, which comprises a single 2D layer of sp^2^‐bonded carbon atoms arranged in a hexagonal honeycomb lattice, is characterized by exceptional transport (thermal and electrical conductivities) and mechanical properties.^[^ ^27^, ^28^, ^29^ ^]^ A thin stack of graphene sheets, <100 nm thick, forms graphene nanoplatelets (GnP)^[^ ^22^ ^]^ with typical TC values of up to ≈3000 W m^−1^ K^−1^.^[^ ^30^ ^]^ Graphite comprises an infinite 3D crystal consisting of thick stacked graphene layers.^[^ ^31^ ^]^ The TC enhancement of the composite material, versus the original molten salt, depends not only on the intrinsic TC of the fillers but also on their dispersion quality in the matrix, which is affected by the homogeneity of the composite, filler–matrix segregation, and filler aggregation.^[^ ^32^, ^33^ ^]^ Therefore, for TC enhancement, a GnP filler is superior to graphene (even though graphene has a higher intrinsic TC), because graphene has a tendency to aggregate, resulting in poor dispersion quality and hence low TC enhancement.^[^ ^34^ ^]^ It has been shown that the intrinsic TC of large‐sized GnPs is higher than that of smaller GnPs, and therefore loading a matrix with large GnPs is preferred.^[^ ^24^, ^35^, ^36^, ^37^ ^]^ Moreover, in a system loaded with smaller GnPs, there are more filler‐to‐filler and filler‐to‐matrix interactions and a higher concentration of filler edges, resulting in increased phonon scattering and hence reduced overall composite TC,^[^ ^38^ ^]^ as may be expected in light of the dominant effect of phonon transport in the TC of graphene‐based allotropes.^[^ ^39^, ^40^, ^41^ ^]^ It is now known that loading a matrix with as‐produced GnP typically results in aggregation and precipitation,^[^ ^34^ ^]^ which reduces the TC enhancement. Better TC enhancement can be achieved by improving the dispersion quality;^[^ ^25^, ^26^, ^40^, ^42^ ^]^ for example, it has been shown that the good dispersion quality of GnP fillers in a polymer matrix allows the formation of a filler network that further enhances the TC.^[^ ^24^, ^36^ ^]^

**Figure 1:**
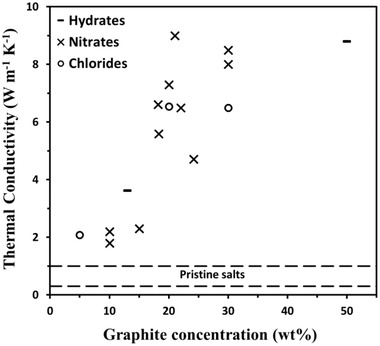
Thermal conductivity of various graphite‐loaded salt composites versus graphite concentration. The TC range of the pristine salts is ≈0.3–1 W m^−1^ K^−1^ (dashed lines).^[^ ^55^, ^56^, ^57^, ^58^, ^59^, ^60^ ^]^

The low TC values of pristine salts (< 1 W m^−1^ K^−1^, **Figure** **1**) have been enhanced by various methods, yielding TC values ranging from 0.2–3 W m^−1^ K^−1^ for ceramic‐based systems^[^ ^43^, ^44^, ^45^, ^46^, ^47^, ^48^, ^49^, ^50^, ^51^, ^52^, ^53^, ^54^ ^]^ and up to 9 W m^−1^ K^−1^ for carbon‐based systems (Figure 1 and Table S1, Supporting Information).^[^ ^55^, ^56^, ^57^, ^58^, ^59^, ^60^ ^]^ As expected, an increase in the concentration of filler (graphite in this case) was found to enhance the TC of the salt composite (Figure 1). However, high filler loading has two disadvantages: it is expensive and it reduces heat capacity and heat of fusion in SHS and LHS applications.^[^ ^61^, ^62^, ^63^ ^]^ It should be noted that the reported TC enhancements in Figure 1 were achieved by different composite preparation methods, such as filler impregnation in molten salt and stirring^[^ ^57^, ^59^, ^60^ ^]^ or dissolution in water and evaporation.^[^ ^55^, ^56^, ^58^ ^]^ These preparation methods involve mechanical forces or an oxidizing environment that could affect the filler's properties (e.g., by reducing its size or increasing defect density) and will consequently reduce the TC enhancement.

Combining more than one type of filler (and optimizing the ratio of the two fillers) could produce a synergistic effect by forming a more efficient percolating network with significantly reduced thermal interface resistance.^[^ ^42^ ^]^ Specifically, combining micron‐size and nano‐size fillers is beneficial for TC enhancement, since small nanofillers could bridge the gaps between the large‐sized particles. The bridging will increase the contact area between the fillers and reduce the filler edge concentration (where a high filler edge concentration would reduce the TC due to phonon scattering).^[^ ^25^, ^64^ ^]^

It has been shown that the improved dispersion quality of graphitic materials^[^ ^65^ ^]^ in molten salts can be attributed to the impregnation of salt ions between the graphitic layers^[^ ^66^, ^67^ ^]^ and that molten salts thus constitute a suitable medium for the exfoliation of graphite to GnP.^[^ ^68^, ^69^ ^]^ In such a system, exfoliation is achieved due to the high wettability of the molten salt on the graphite surface,^[^ ^68^, ^69^, ^70^, ^71^, ^72^, ^73^ ^]^ facilitating its impregnation within the graphite layers.^[^ ^69^ ^]^ This impregnation enables the propagation of a holes‐rich melt in the interlayer spacings, generating gentle shear forces that exfoliate the graphite to GnP.^[^ ^68^ ^]^

In this study, we demonstrated the application of the above‐described approach for the enhancement of salt TC values (for subsequent application in TES systems). The novelty of our methodology lies in the formation of a *hybrid* graphite–GnP filler *directly* in the molten salt by partial exfoliation of the graphite to GnP (in comparison to other techniques, based on a single filler or a pre‐prepared hybrid filler).^[^ ^55^, ^56^, ^57^, ^58^, ^59^, ^60^ ^]^ The methodology applied in this study provides a TC enhancement in the composite material versus the pristine salt of two orders of magnitude (0.64 W m^−1^ K^−1^ to 44 W m^−1^ K^−1^), due to good dispersion of the fillers in the matrix and the bridging between large graphite particles by smaller GnP particles. Furthermore, this study provides a jumping‐off point for the potential application of the molten salt‐graphite system in TES, since it reveals some of the characteristics and behaviors of salt‐graphite composites.

## 2 Results and Discussion

The TC of a eutectic NaCl‐KCl salt mixture (often used in TES applications)^[^ ^74^ ^]^ was enhanced by loading it with graphite flakes (GF), which were then partially exfoliated to GnPs by thermal treatment (tt), i.e., 800 °C for 2 h. The composite so formed (*see* Experimental Section) consisted of thermally treated GF (GF_tt_) and GnP (GnP_tt_), both impregnated by the salt. The TC values of GF–salt and GnP–salt mixtures without thermal treatment were also obtained and served as reference values.

### 2.1 Dispersion of the Carbon‐Based Fillers

**Figure 2:**
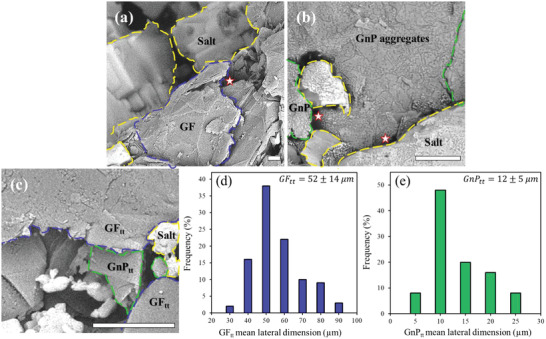
a,b) SEM imaging (BSE mode) of (a) GF–salt mixture (without thermal treatment) and b) GnP–salt mixture (without thermal treatment), characterized by multiple voids (stars) and aggregates. c) SEM imaging of GF_tt_ in molten salt, which resulted in partial exfoliation of GF_tt_ to GnP_tt_. The GnP_tt_ produced in the hybrid composite so formed bridged between the large GF_tt_ particles, thereby decreasing the thermal interface resistance. d,e) Distribution histograms of the mean lateral dimensions of GF_tt_ (d; ≈50 µm) and GnP_tt_ (e; ≈10 µm), as extracted from SEM micrographs (> 100 particles). The concentration of carbon‐based fillers in all samples was 23 wt.%. GF and GnP were characterized by a layered morphology (blue and green dashed lines, respectively), and segregated salt was characterized by bright regions (yellow dashed line). Scale bar = 10 µm.

As mentioned above, the TC of composite materials is dictated mainly by the filler concentration, morphology, and dispersion quality in the matrix.^[^ ^24^, ^75^, ^76^ ^]^ The last of these factors is especially relevant for carbon‐based fillers, which tend to aggregate and precipitate, giving composites with low TC values.^[^ ^34^, ^77^ ^]^ The dispersion quality of the various composites prepared for this study was investigated by SEM imaging (**Figure** **2**). The first reference system, namely, a GF–salt mixture, showed large GF particles in the vicinity of large salt aggregates (Figure 2a), with poor GF‐GF and GF‐salt contacts (i.e., multiple voids, indicated by stars), which means poor dispersion quality of the filler particles in the matrix. The second reference system, the GnP–salt mixture showed even worse dispersion quality, since the small GnP particles formed GnP aggregates as large as the GF particles (≈50 µm in size, Figure 2b) but with higher filler‐filler interface thermal resistance than GF. Moreover, the GnP aggregation was followed by the formation of segregated salt regions (Figure 2b). In contrast, thermal treatment of GF in a molten salt (hybrid system, Figure 2c) facilitated the impregnation of salt in between the GF_tt_ layers (≈50 µm in size, Figure 2d), resulting in partial exfoliation of GF to GnP.^[^ ^68^, ^69^ ^]^ The exfoliated GF_tt_ (i.e., GnP_tt_, ≈10 µm in size, Figure 2e) particles did not aggregate, but bridged the filler‐filler and filler‐matrix interface, reducing the volume of voids (Figure 2c). Therefore, in this system, better dispersion quality was achieved, as can also be observed in SEM micrographs (Figure S1, Supporting Information).

As mentioned above, for TES applications a high TC of the composite is a major requirement for efficient heat dissipation.^[^ ^17^, ^21^ ^]^ Just as poor dispersion quality of the filler (GF and GnP, Figure 2a,b) is expected to lead to a high thermal interface resistance at the filler‐matrix interface, so, too, is the formation of a network of fillers expected to enhance the TC of the salt composite.^[^ ^64^, ^78^ ^]^ We suggest that the thermal treatment of GF in molten salt resulted in partial exfoliation to GnP and the formation of a high TC GF_tt_–GnP_tt_ network in the hybrid salt composite, thereby enhancing the TC of the composite.

### 2.2 Hybrid GF_tt_‐GnP_tt_ Composite

**Figure 3:**
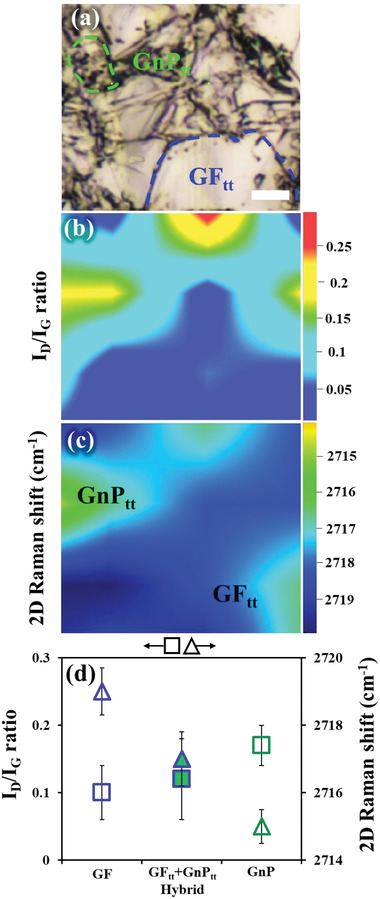
a–c) Raman imaging of the hybrid GF_tt_–GnP_tt_ (after salt removal). a) The imaged area (≈100 µm × 100 µm) was mapped according to b) the defect density (*I_D_/I_G_* ratio) and c) the number of layers (2D Raman shift). d) Mean values (see Experimental section) of the *I_D_/I_G_* ratio (left ordinate) and 2D peak (right ordinate) of as‐received GF (blue) and GnP (green) powders and the thermally treated hybrid (GF_tt_–GnP_tt_). The concentration of carbon‐based fillers in all samples was 23 wt.%. Scale bar = 10 µm.

The locations of the large GF_tt_ particles and smaller GnP_tt_ particles in the hybrid GF_tt_–GnP_tt_ salt composite (**Figure** **3**a) were mapped by Raman spectroscopy in terms of their defect density (*I_D_/I_G_* ratio, Figure 3b), and the number of layers (2D bands, Figure 3c). By comparing the imaging mode (Figure 3a) to the mapping modes (Figure 3b and 3c), we found both *large GF_tt_* particles (bright region, Figure 3a) and *small* *GnP*_*tt*_ particle*s* (a dark region in Figure 3a), as detailed below. The GF_tt_ particles were characterized by a low *I_D_/I_G_* ratio (< 0.1, blue area in Figure 3b) and a 2D band of > 2719 cm^−1[^ ^79^ ^]^ (*see* Experimental section and blue area in Figure 3c), while the GnP_tt_ particles were characterized by a higher *I_D_/I_G_* ratio (> 0.1,^[^ ^80^, ^81^ ^]^ green areas in Figure 3b) and a 2D band of 2714–2719 cm^−1[^ ^82^, ^83^ ^]^ (green area in Figure 3c). The observed Raman spectrum agrees with previously reported data.^[^ ^80^, ^81^, ^82^, ^83^ ^]^

The *I_D_/I_G_* mapping (Figure 3b) indicated the coexistence of *large* *GF_tt_* particles (≈50 µm in size, Figure 2d), with a low edge defect density, and exfoliated *small* *GnP_tt_* particles (≈10 µm in size, Figure 2e), with a higher edge defect density,^[^ ^84^, ^85^ ^]^ in agreement with the SEM results (Figure 2c). As expected, the relatively smaller GnP_tt_ particles had a higher edge concentration than the GF_tt_ particles and, consequently, a higher *I_D_/I_G_* ratio.^[^ ^86^ ^]^ The 2D mapping (Figure 3c) showed the locations of the large GF_tt_ particles (2D > 2719 cm^−1^, blue area in Figure 3c) and the small GnP_tt_ particles (2714‐2719 cm^−1^, green in Figure 3c). The shift to a lower 2D wave number indicated a decrease in the number of layers.^[^ ^81^, ^82^, ^83^ ^]^ These results are in line with the particle sizes of the GF_tt_ and GnP_tt_ obtained by *I_D_/I_G_* mapping (Figure 3b) and SEM imaging (Figure 2d,e).

**Figure 4:**
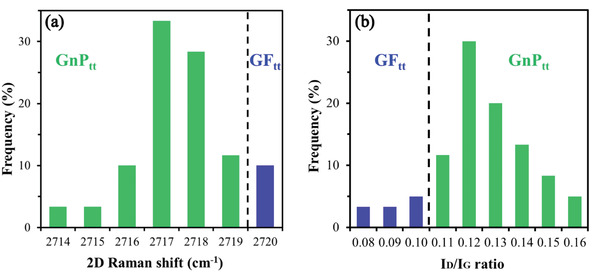
Exfoliation yield of molten salt–GF_tt_ measured at the atomic scale: distributions of a) 2D Raman shifts and b) *I_D_/I_G_* ratio (*n* = 60 measured points in an area of ≈100 µm × 100 µm) indicate a composition of 10% GF_tt_ (blue) and 90% GnP_tt_ (green), i.e., an exfoliation yield of ≈90%.

Overall, the 2D Raman shift mapping agreed with the *I_D_/I_G_* mapping, and both demonstrated a clear difference between GF_tt_ and GnP_tt_ particles in terms of defect density, number of layers, and size, in line with the SEM imaging (Figure 2c). The mean values of the 2D Raman shift and the *I_D_/I_G_* ratio (Figure 3d) of the hybrid composite (i.e., GF_tt_+GnP_tt_) were extracted from their distribution histograms (see Experimental Section, **Figure** **4**a,b, respectively). These values lie between those of the raw materials, GF (blue) and GnP (green) powders (Figure 3d; Figures S2 and S3, Supporting Information), demonstrating the partial exfoliation of the starting GF to GnP.

In addition, the exfoliation yield of GF_tt_ was calculated from the 2D Raman shift distribution (Figure 4a) by segmenting the histogram into GnP_tt_ (2714‐2719 cm^−1^) and GF_tt_ (> 2719 cm^−1^).^[^ ^87^, ^88^, ^89^ ^]^ This procedure showed that only 10% of the GF_tt_ particles were not exfoliated to GnP_tt_ during the thermal treatment, i.e., the exfoliation yield of GF_tt_ to GnP_tt_ was ≈90% (Figure 4a). The same exfoliation yield was also extracted from the *I_D_/I_G_* ratio distribution (Figure 4b), where the GnP_tt_ particles were characterized by an *I_D_/I_G_* ratio > 0.10, which is the mean *I_D_/I_G_* ratio of as‐received GF (Figure 4d).

**Figure 5:**
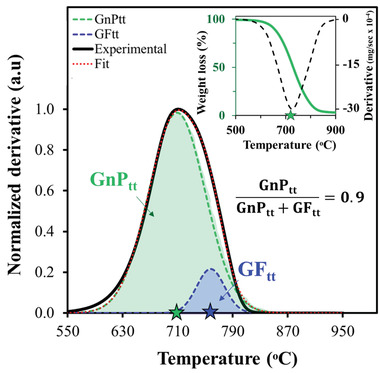
The exfoliation yield of molten salt–GF_tt_ measured at the macroscopic scale: deconvolution of TGA normalized derivative peak (black curve) into two peaks of GF_tt_ (blue) and GnP_tt_ (green), according to the *T_1/2_* of the as‐received GF (≈760 °C, blue star) and GnP (≈710 °C, green star), showing a GnP_tt_‐to‐(GnP_tt_+GF_tt_) ratio of 0.9 (i.e., 90% exfoliation yield). Inset: A typical thermogram (weight loss, green) and its first derivative (dashed black curve). The temperature at which the GnP combustion reaches half of its total weight loss (star) is defined as *T_1/2_* (710 °C).

To provide support for the Raman findings (atomic scale), the exfoliation yield was also estimated by thermogravimetric analysis (TGA; macroscopic scale), i.e., in terms of the mean combustion temperature at which one‐half of the total weight loss is reached (*T_1/2_*; **Figure** **5**, inset).^[^ ^80^ ^]^ The typical *T_1/2_* values of as‐received GF and GnP are ≈760 °C and ≈710 °C (Figure 5, blue and green stars, respectively), in agreement with previous studies.^[^ ^80^, ^88^ ^]^ We found that *T_1/2_* values can be used to differentiate between GF_tt_ and GnP_tt_ in the hybrid composite since higher values of *T_1/2_* indicate a larger GF_tt_/GnP_tt_ ratio.^[^ ^80^, ^89^ ^]^ Therefore, we deconvoluted the normalized derivative of a TGA peak of the hybrid composite (Figure 5, black curve) into two separate GF_tt_ and GnP_tt_ peaks (Figure 5, blue and green areas, respectively), according to the *T_1/2_* values of the as‐received GF and GnP (≈760 °C and ≈710 °C, Figure 5, blue and green stars). The exfoliation yield was then calculated as the ratio of the area under the GnP_tt_ peak to the total area (GnP_tt_+GF_tt_ peaks, see Experimental section) in the TGA (bulk measurement, Figure 5), i.e., ≈90%, in line with the Raman imaging results (atomic scale measurement).

Below, we discuss the TC enhancement in the hybrid composite and how it is affected by filler type, concentration and dispersion quality, and the formation of a thermally conductive network.

### 2.3 TC of the Composite

**Figure 6:**
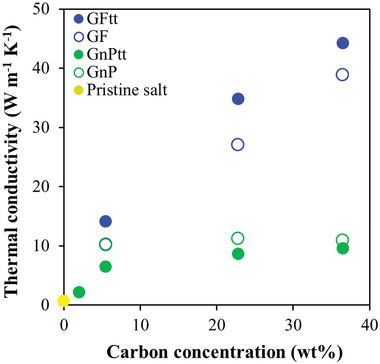
TC of the pristine salt (yellow) loaded with GnP (green) or GF (blue) filler versus filler concentration. Thermally treated (tt) and untreated samples are denoted by full and empty symbols, respectively.

Mixing either of the carbon‐based fillers (GF or GnP) with the molten salt or thermally treating GF in the salt enhanced the TC value of the salt composite compared to the pristine salt (**Figure** **6**). The TC increase in the GnP–salt reference system, versus the salt alone, was rather moderate (from 0.64 to 10 W m^−1^ K^−1^), with no substantial effect of thermal treatment or GnP concentration (Figure 6). However, in the GF–salt reference mixture, the effect of the filler on the TC was strongly concentration‐dependent, yielding a TC of 39 W m^−1^ K^−1^ at 36 wt.% GF (Figure 6). These results indicate significantly higher TC enhancement compared to previous reports (up to 9 W m^‐1^ K^−1^ at 21 wt.% of graphite, Figure 1); this “extra improvement” may be attributed to the composite preparation method in this study, which does not involve mechanical forces or an oxidizing environment and hence does not downgrade the quality of the fillers.^[^ ^90^ ^]^ The thermally treated GF (GF_tt_–GnP_tt_–salt hybrid composite) yielded the highest TC value (44 W m^−1^ K^−1^ at 36 wt.% GF, Figure 6), which was ≈30% higher than that of the GF‐salt reference mixture (GF_tt_ versus GF, Figure 6).

Generally, the GnP‐based systems were characterized by lower TC values than the GF‐based systems (green and blue in Figure 6, respectively), despite the higher intrinsic TC value of the former (≈3000 vs ≈1000 W m^−1^ K^−1^, respectively).^[^ ^21^, ^30^ ^]^ A possible reason for this finding is the better dispersion quality of the GF‐based mixtures in the salt compared to the GnP‐based mixtures, which tend to aggregate (Figure 2b).

The good dispersion quality (as in the GF_tt_ system, Figure 2c) led to the formation of a network of fillers, and, consequently, the TC of the composite was two orders of magnitude higher than that of the pristine salt (44 versus 0.64 W m^−1^ K^−1^). It was also higher than the TC values of the untreated GnP–salt or GF–salt mixtures (Figure 6). The highest enhancement – obtained for the hybrid system – was associated with the partial exfoliation of the GF_tt_ to GnP_tt_ (Figures 4 and 5), whose good dispersion quality in the salt matrix (Figure 2c) facilitated the formation of a thermally conductive network. In contrast, the GnP_tt_ system demonstrated lower TC values than the GnP–salt mixture, since the treatment resulted in salt segregation and GnP_tt_ aggregation (Figure S4, Supporting Information), due to insignificant salt impregnation into the GnP_tt_. Therefore, poor dispersion quality of the filler was obtained, resulting in the lowest TC enhancement (Figure 6).

### 2.4 Latent Heat of the Composite

**Figure 7:**
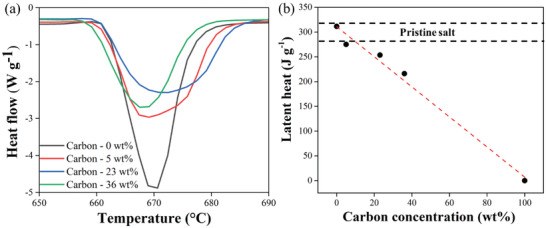
a) DSC curves of the GF_tt_–GnP_tt_–salt composite loaded with various carbon concentrations. b) The latent heat (as calculated from (a)) versus carbon concentration.

For practical TES applications, the latent heat of phase change composite is an important parameter. To determine this parameter, differential scanning calorimetry (DSC) measurements (heating and cooling) were obtained in temperature range 500–800 °C (**Figure** **7**a and Figure S6, Supporting Information). The fusion enthalpies of the GF_tt_‐GnP_tt_‐salt samples with different carbon concentrations were extracted from the DSC curves (Figure 7b and Table S2, Supporting Information). The measured latent heat energy of the pristine salt was 309.39±5.27 J g^−1^, in agreement with the literature.^[^ ^91^, ^92^ ^]^ With increasing carbon concentration, a linear decrease in the latent heat was found, as expected (Figure 7b and Table S2, Supporting Information).^[^ ^59^, ^60^, ^91^, ^92^ ^]^ Moreover, repeating the heating/cooling procedure 100 times (cycling test) indicated that the onset of the melting temperature of the GF_tt_‐GnP_tt_‐salt composite was completely preserved during the phase change cycling (Figure S7, Supporting Information). It also suggests that carbon and salt are chemically inert to each other.

In summary, we suggest that the GnP_tt_ particles (partially exfoliated from GF during thermal treatment, 90% conversion efficiency, Figures 4 and 5) formed an efficient thermally conductive network that bridged the filler‐filler and filler‐matrix interfaces (Figure 2c) and consequently enhanced the TC of the composite material (Figure 6). This did not occur after mixing either GF or GnP with the salt at room temperature (without thermal treatment) due to the aggregation of the fillers (Figure 2a,b). The GF_tt_ system (i.e., the hybrid system) consisted of 10 wt% large GF_tt_ particles (blue, Figure 2c) connected by small GnP_tt_ particles (green, Figure 2c), indicating a synergistic 3D heat conduction network (dashed line, Figure S5, Supporting Information). This network provided additional heat flow routes (compared to the GF–salt or GnP–salt mixtures) and reduced the thermal interface resistance.

## 3 Conclusions

A thermally conductive salt composite was obtained by loading a NaCl‐KCl salt mixture with GF and then applying thermal treatment, which facilitated the partial exfoliation of GF to GnP. The resulting hybrid salt composite was characterized by good dispersion quality of the filler and an enhanced TC (44 W m^−1^ K^−1^). The enhancement was achieved by the formation of a thermally conductive network, where the GnP_tt_ bridged between GF_tt_ particles, thereby decreasing the thermal interface resistance. The TC enhancement of two orders of magnitude (up to ≈44 W m^−1^ K^−1^) compared to the pristine salt matrix (≈0.64 W m^−1^ K^−1^) exceeded that previously reported in the literature.

## 4 Experimental Section

### Materials

Graphite flakes (GF, Asbury Carbons), KCl (Carlo Erba reagents, >99 wt% Cl), NaCl (Frutarom, >99 wt% Cl), ethanol (EMSURE ACS; Analytical grade) and GnP type H‐15 (xG Sciences,^[^ ^93^ ^]^ 15 µm mean lateral size) were used as received.

### Preparation of the GF‐GnP‐Salt Composite

**Figure 8:**
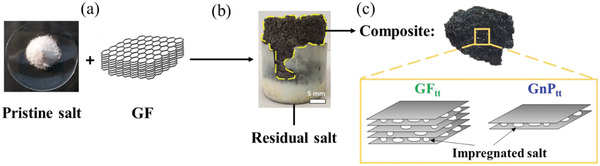
Schematic flowchart of the preparation of the GF–GnP–salt composite: a) Mixing a eutectic NaCl‐KCl mixture and GF. b) Thermal treatment – heating at 5 °C min^−1^ to 800 °C under an inert argon flow (50 mL min^−1^). c) Separation of the composite phase from the residual salt for further characterization.

The methodology for the preparation of the composite comprised three stages: 1) Salt drying – absorbed water was removed from the hygroscopic salt mixture by heating it to 500 °C for 3 h and storing the material at 150 °C until use. 2) Mixing – GF or GnP, at various carbon‐based filler concentrations, was mixed in a mortar with a dry eutectic NaCl‐KCl mixture. 3) Thermal treatment – the mixture was then loaded into an alumina crucible, which was inserted into a vertical tube reactor equipped with a temperature controller and an argon gas inlet and heated at a rate of 5 °C min^−1^ under an argon flow (50 mL min^−1^) until the mixture reached 800 °C (above the melting temperature of eutectic NaCl‐KCl, 658 °C).^[^ ^65^ ^]^ The mixture was then held at 800 °C for 2 h. Thereafter, the material was left to cool at room temperature. This procedure is referred to as “thermal treatment” (tt) in this article. At the end of the thermal treatment process, the upper phase (designated the “composite”; see **Figure** **8**) was separated off and used for all the experiments. The composite phase (circled in yellow in Figure 8) consisted of thermally treated graphite (GF_tt_) and exfoliated GnP (GnP_tt_), both impregnated by the salt.

For further characterization, the composite phase was separated from the impregnated salt by washing with hot water (80 °C, 250 mL) and vacuum filtration (Sartorius, 0.2‐µm pores), followed by drying at 80 °C for 24 h. The remaining material (salt‐free GF_tt_ + GnP_tt_) was weighed to calculate the carbon concentration in the composite (**Table** **1**).

**Table 1:** Initial GF concentration in the GF–salt mixture before thermal treatment and salt‐free GF_tt_ + GnP_tt_ concentration in the obtained composite

Initial GF concentration [wt.%]	GF_tt_+GnP_tt_ concentration in the composite [wt.%]
2	5
10	23
20	36

Due to the relatively low content of salt impregnated into the GF_tt_ interlayers in the composite,^[^ ^68^, ^69^, ^94^ ^]^ a relatively high volume of residual salt was formed (Figure 8). Therefore, the carbon concentration in the composite was higher than its initial concentration in the original mixture (Table 1). The maximum initial GF concentration was limited to 20 wt% (Table 1), in line with previous studies,^[^ ^78^, ^95^ ^]^ due to the trade‐off between TC (increases with GF addition) and energy storage capacity in the salt (decreases with GF addition).^[^ ^61^, ^62^, ^63^ ^]^ Moreover, increasing the GF concentration promotes aggregation and precipitation of the filler and, consequently, poor dispersion quality.^[^ ^96^, ^97^ ^]^

### Characterization Techniques

Raman spectroscopy was used to characterize the defect density and to identify the layered materials (GF or GnP). A Horiba Jobin Yvon HR LabRAM micro‐Raman device was operated at 532 nm, with a laser spot size of 1 µm (the powdered sample was placed on a quartz slide). The Raman spectra were characterized by:^[^ ^98^ ^]^ i) a G band (≈1580 cm^−1^) – indicates the defect density, ii) a 2D band (≈2700 cm^−1^) – indicates the number of layers, and iii) a D band (≈1350 cm^−1^) – indicates in‐plane and edge defects.^[^ ^98^ ^]^

Raman mapping^[^ ^99^, ^100^ ^]^ of the 2D Raman shift and the *I_D_/I_G_* ratio, indicating the number of layers and the defect density, respectively, provided the locations of the GF_tt_ and GnP_tt_ particles (an area of ≈100 µm × 100 µm was scanned), from which the yield of the exfoliation process of GF to GnP could be calculated. The mean values of the 2D Raman shift and *I_D_/I_G_* ratio were extracted from their distribution histograms (*n* = 60 measured points) to compare the properties of GF_tt_ and GnP_tt_ with those of the as‐received GF and GnP. A 2D shift of 2719 cm^−1[^ ^87^, ^88^, ^89^ ^]^ was used as a limit between GnP and GF.

Thermogravimetric analysis (TGA) was performed in a Mettler Toledo analyzer with a Stare software system (TGA/STDA85). Samples of 3−6 mg were placed in 70‐µL alumina crucibles, heated under an air flow (50 mL min^‐1^) to 500 °C at a rate of 10 °C min^−1^ and then from 500 to 1000°C at 5°C min^−1^. Then, the temperature was kept at 1000 °C for 30 min. The thermograms were used to determine the GnP:GF ratio in the composite by analyzing the mean combustion temperature (termed *T_1/2_*), at which half of the total weight was lost,^[^ ^80^ ^]^ where 630−750 °C and 830−1000 °C are typical temperature ranges for GnP and GF decomposition, respectively.^[^ ^80^ ^]^ The *T_1/2_* values were extracted from the obtained peaks in the curves of the TGA first derivative, differentiating GnP from GF. The ratio of the areas under these peaks corresponds to the GnP‐to‐GF concentration ratio. The deconvolution was performed using Origin software.

Differential scanning calorimetry (DSC) was performed with a Labsys Evo thermal analyzer (Setaram, Caluire, France), equipped with a 3D‐Cp heat capacity sensor. The samples (30 mg) were loaded in 380 µL Pt crucibles that included lids and measured in Ar atmosphere (flow rate – 40 mL min^−1^). Sample temperature calibration was performed by melting standards of Sn, Ag, and Au in alumina crucibles before every run. Each sample was heated to 500 °C (30 °C min^−1^) and then a heating and cooling program was applied to each sample and repeated twice to ensure consistency. This program comprised holding the sample at 500 °C for 5 min, heating to 800 °C (10°C min^−1^), maintaining the temperature at 800 °C for another 5 min, and cooling down to 500 °C (10 °C min^−1^). Each measurement was repeated 4 times (average error of <3%). For the cycling test, the heating/cooling procedure was repeated 100 times.

Scanning electron microscopy (SEM) was used to investigate the dispersion quality of the carbon‐based fillers in the salt. A high‐resolution cold field emission gun Scanning Electron Microscopy (SEM, JSM‐7400F, JEOL) was operated in backscattered electron (BSE) mode. The samples were prepared by spreading the composite over a strip of sticky carbon tape mounted on an aluminum disk. The samples were then carbon‐coated (≈5 nm, carbon fiber flash deposition, K575X, EMITECK) to increase their electrical conductivity.

Thermal conductivity was measured using a thermal analyzer (TPS 2500s, Hot Disk, Sweden) based on the transient plane source (TPS) method.^[^ ^101^ ^]^ Pristine salt and salt composites were compressed (200 bars, hydraulic press, APEX, A‐14, UK) to form pellets (25 mm in diameter and 6 mm in thickness) on which the TC measurements were conducted (in air at 25 °C). Each measurement was repeated five times, providing reproducible values with an average error of ≈0.001 W m^−1^ K^−1^.

## Conflict of Interest

The authors declare no conflict of interest.

## Supporting information

Supporting Information

## Data Availability

The data that support the findings of this study are available from the corresponding author upon reasonable request.
