# Supplementary material for: Thermally Conductive Molten Salt for Thermal Energy Storage: Synergistic Effect of a Hybrid Graphite‐Graphene Nanoplatelet Filler
Source: Glob Chall. 2023 Aug 31;7(9):2300053. doi: 10.1002/gch2.202300053 (PMC10517311; doi:10.1002/gch2.202300053)
Supplement: Supplementary file 1 — Supporting Information [file GCH2-7-2300053-s001.pdf]

Global Challenges
Open Access

Supporting Information

for *Global Challenges.*, DOI 10.1002/gch2.202300053

Thermally Conductive Molten Salt for Thermal Energy Storage: Synergistic Effect of a Hybrid Graphite-Graphene Nanoplatelet Filler

Adi Lavi, Avia Ohayon-Lavi, Yelena Leibovitch, Shmuel Hayun, Efrat Ruse* and Oren Regev**

Supporting Information

Thermally Conductive Molten Salt for Thermal Energy Storage: Synergistic Effect of a Hybrid Graphite-Graphene Nanoplatelet Filler

Adi Lavi, * *Avia Ohayon-Lavi, Yelena Leibovitch, Shmuel Hayun, Efrat Ruse,* * *and Oren Regev* *

Table S1. Thermal conductivity (TC) of several salts with the addition of graphite in various concentrations

Salt	TC – pristine salt [W m^{-1} K^{-1}]	Graphite concentration [wt%]	TC – composite [W m^{-1} K^{-1}]	Reference
NaCl-$CaCl_2$-$MgCl_2$	1.174	5	2.084	[1]
$NaNO_3$	0.9	10	1.8	[2]
$Na_2SO_4(10H_2O)$-$Na_2HPO_4(12H_2O)$	0.5	13	3.615	[3]
KNO_3	1	15	2.3	[2]
$LiNO_3$-$NaNO_3$	0.84	18.2	6.61	[4]
$LiNO_3$-KCl	0.93	18.3	5.59	[4]
NaCl-$CaCl_2$	0.704	20	6.54	[5]
$NaNO_3$-KNO_3	0.7	21	9	[2]
$LiNO_3$-NaCl	0.8	24.2	4.71	[4]
$CaCl_2(6H_2O)$	0.596	50	8.796	[6]

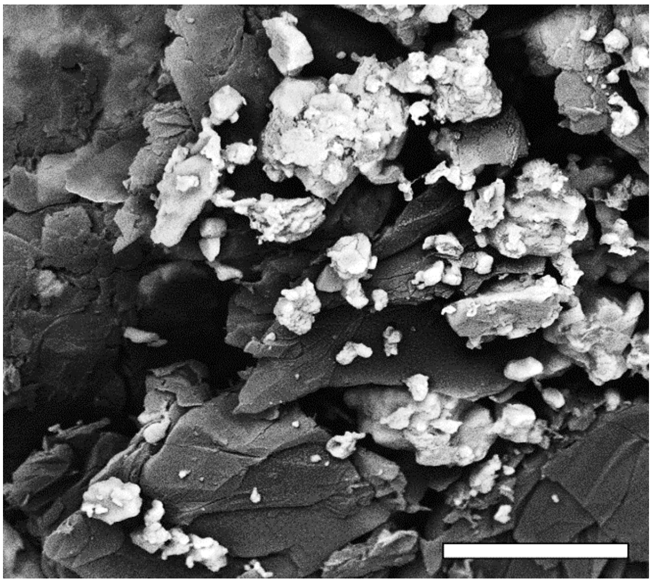

Figure S1. SEM micrograph of the hybrid GF_{tt}–GnP_{tt} (black)–salt (white) composite, showing high dispersion quality of the salt. Scale bar = 20 μm.

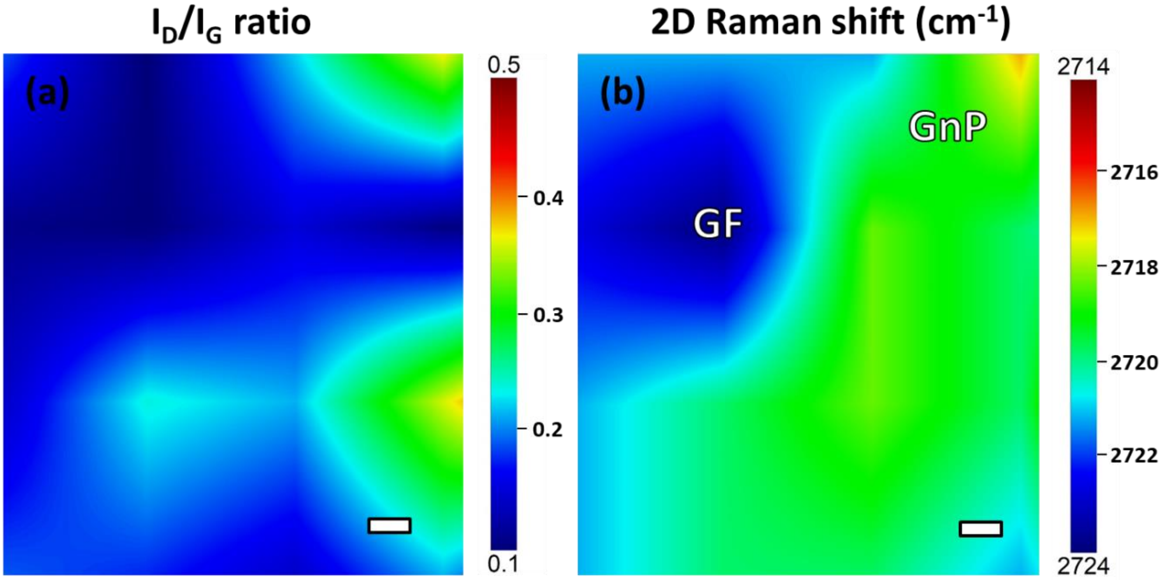

Figure S2. Raman imaging of as-received GF: (a) defect density (I_D/I_G ratio) and (b) number of layers (2D Raman shift). Scale bar = 5 μm.

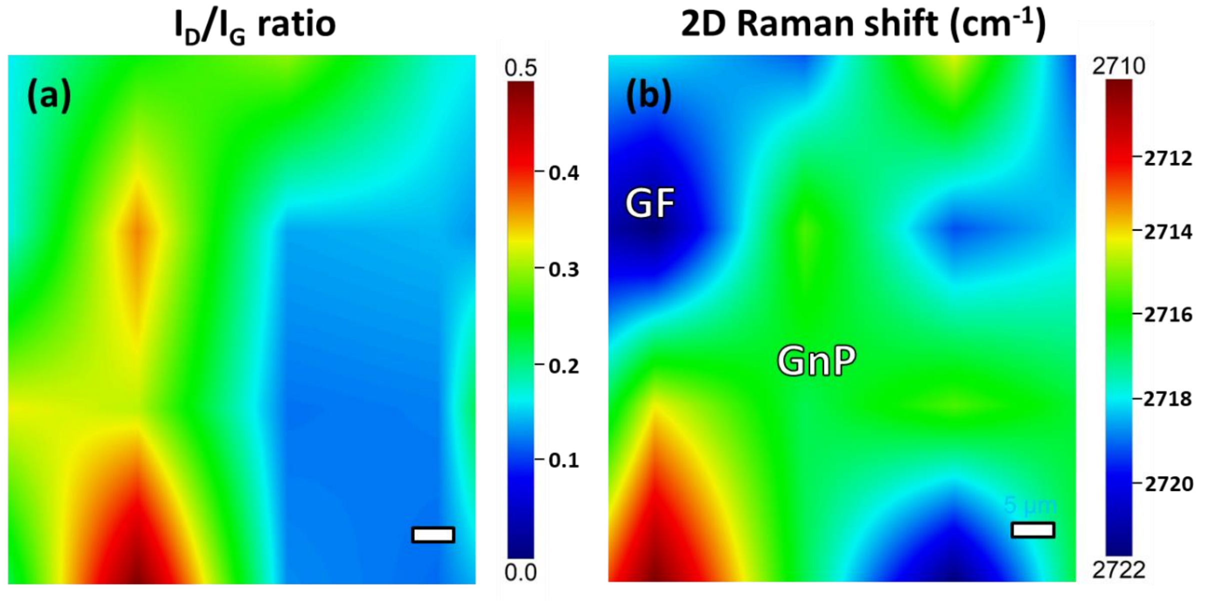

Figure S3. Raman imaging of as-received GnP: (a) defects density (I_D/I_G ratio) and (b) number of layers (2D Raman shift). Scale bar = 5 μm

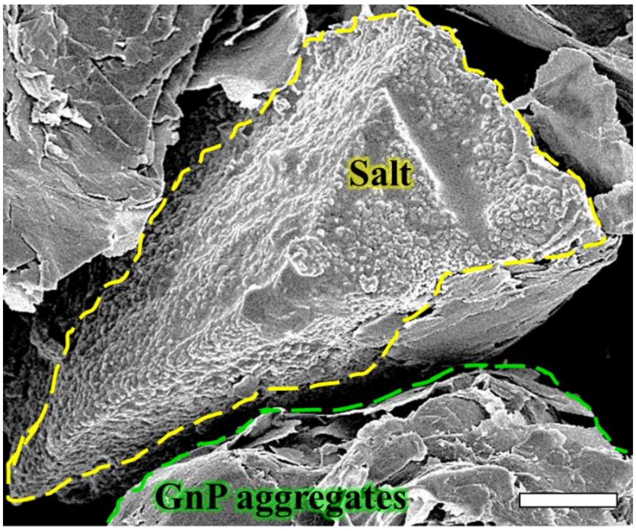

Figure S4. SEM micrograph of GnP_{tt} system showing salt segregation (yellow) and GnP_{tt} aggregation (green) due to insignificant salt impregnation, which results in poor dispersion quality and low TC enhancement. GnP_{tt} concentration is 23 wt%. Scale bar = 10 μm.

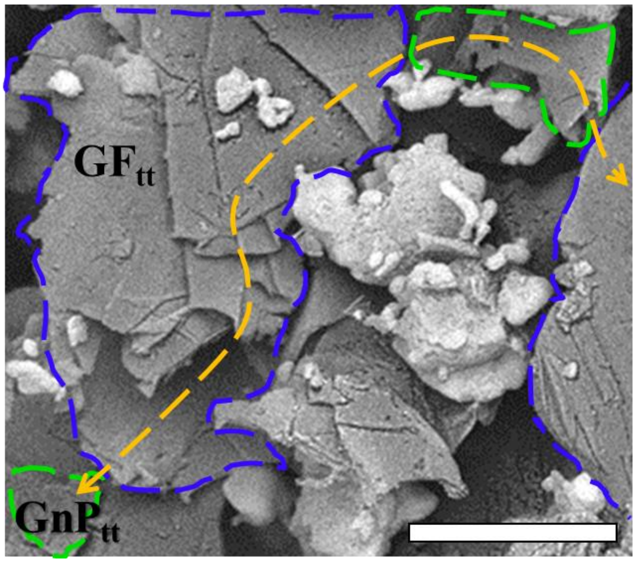

Figure S5. SEM micrograph demonstrating the percolation pathway (yellow line) in the hybrid GF_{tt}–GnP_{tt}–salt composite, formed via bridging of the GF_{tt} particles (blue) with GnP_{tt} (green) particles. Salt particles are white. Scale bar: 10 μm.

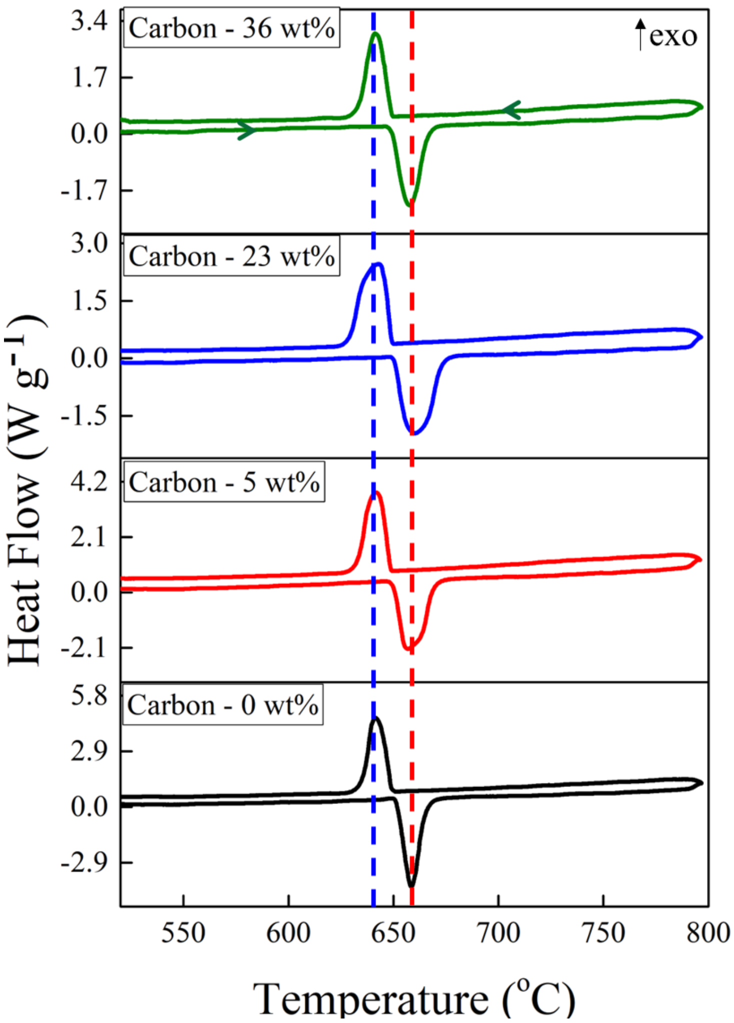

Figure S6. Differential scanning calorimetry (DSC) heating and cooling curves of the GF_{tt}-GnP_{tt}-salt samples at various carbon concentrations. Heating and cooling peaks are indicated by the red and blue dashed lines, respectively.

Table S2. Latent heat of the GF_{tt}-GnP_{tt}-salt samples loaded with various carbon concentrations, as measured by DSC.

Carbon concentration [wt%]	Latent Heat [J g^{-1}]
0	309.39 ± 5.27
5	275.21 ± 3.16
23	247.42 ± 4.06
36	211.00 ± 4.86

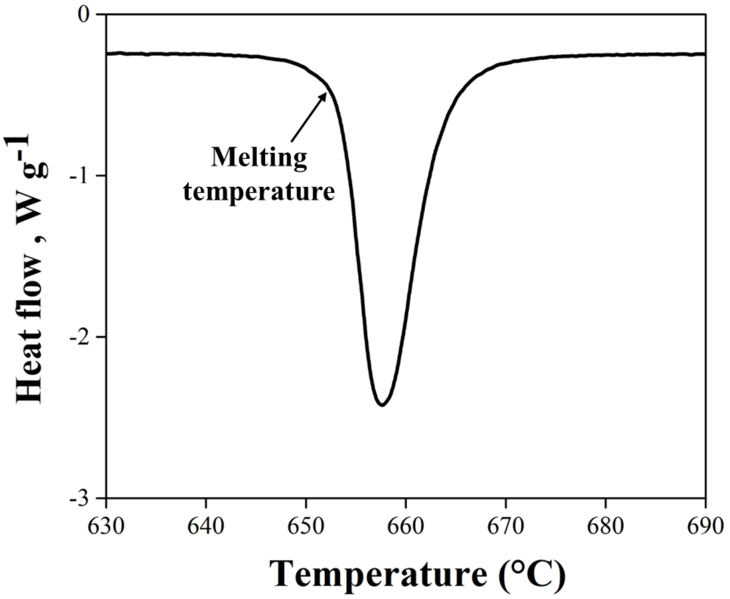

Figure S7. DSC curve of the GF_{tt}–GnP_{tt}–salt composite loaded with 36 wt% carbon, after 100 heating/cooling cycles (500-800 °C), indicating stable melting temperature and that the salt and carbon are chemically inert to each other.

References

[1] H. Tian, W. Wang, J. Ding, X. Wei, M. Song, J. Yang, *Appl. Energy* **2015**, *148*, 87.

[2] S. Pincemin, R. Olives, X. Py, M. Christ, *Sol. Energy Mater. Sol. Cells* **2008**, *92*, 603.

[3] Y. Wu, T. Wang, *Energy Convers. Manag.* **2015**, *101*, 164.

[4] L. Zhong, X. Zhang, Y. Luan, G. Wang, Y. Feng, D. Feng, *Sol. Energy* **2014**, *107*, 63.

[5] H. Tian, W. Wang, J. Ding, X. Wei, C. Huang, *Sol. Energy Mater. Sol. Cells* **2016**, *149*, 187.

[6] Z. J. Duan, H. Z. Zhang, L. X. Sun, Z. Cao, F. Xu, Y. J. Zou, H. L. Chu, S. J. Qiu, C. L. Xiang, H. Y. Zhou, *J. Therm. Anal. Calorim.* **2014**, *115*, 111.
